# An Overlooked Hazard for Operating Room Nurses: Ergonomic Risks and Consequences Related to Working Position

**DOI:** 10.1155/nrp/7808208

**Published:** 2025-05-22

**Authors:** Nermin Ocaktan, Ukke Karabacak

**Affiliations:** Faculty of Health Sciences, Department of Nursing, Acıbadem Mehmet Ali Aydinlar University, Istanbul, Türkiye

**Keywords:** ergonomic risk, ergonomics, operating room, operating room nurse, working position

## Abstract

**Background:** A working environment designed for employee comfort and safety reduces occupational risks. In hospitals, the health of service providers should be protected as much as the patients' health. During care practices, nurses interact with their environment and the equipment as well as the patients. Protecting nurses' health and welfare in the working environment is related to the protection of individual, family, and community health. The study was conducted to determine the risks of operating room nurses' body postures and working conditions in the operating room in terms of musculoskeletal disorders and the effects of these risks on nurses.

**Method:** This descriptive study examined the ergonomic risk assessments of operating room nurses' intraoperative positions and their musculoskeletal problems. The “Nurse Introductory Characteristics Form,” “Extended Nordic Musculoskeletal Questionnaire,” and “Rapid Entire Body Assessment Tool” were used to collect data.

**Results:** It was determined that the intraoperative body positions of nurses carry a medium-high level of ergonomic risk; they mostly experience musculoskeletal disorders between the ages of 23.5 and 25.03, and the most affected regions are the neck and lower back. A significant difference was determined between the incidence of musculoskeletal disorders and total working time, working style, receiving undergraduate ergonomics training, and participating in regular sports (*p* < 0.05). An increase in working years increased the risk, while receiving undergraduate ergonomics training decreased it.

**Conclusions:** Nurse-specific recommendations should be developed to prevent ergonomic risks in the operating room and their negative consequences. Undergraduate training including these recommendations is effective in developing positive behaviors regarding the management of ergonomic risks.

## 1. Introduction

The health of service providers should be protected as well as the health of patients who apply for services in healthcare institutions. Healthcare professionals may face potential risks arising from physical, biological, ergonomic, chemical, psychological, and social security aspects of the working environment. Having an unhealthy working environment significantly increases occupational accidents and possible risks, occupational diseases, related disabilities, and even deaths of healthcare professionals [1, 2].

Operating theater nurses are indispensable and critical members of the surgical team. Therefore, they are exposed to various ergonomic risk factors arising from the working environment and their work. These risk factors may negatively affect the physiological and psychological well-being and work performance of operating theater nurses. Although there are a limited number of studies in the current literature examining the exposure of operating theater nurses to ergonomic risk factors and their results, the common results obtained from studies conducted in different disciplines and in the field of nursing are that these risks can be grouped as physical, chemical, psychosocial, and biomechanical risks [3, 4].

Among the physical ergonomic risk factors related to the work performed in the operating theater, standing for a long time, remaining fixed in the same position, limited movement, and repetitive movements have an important place. In addition, conditions such as inadequate lighting and inappropriate temperature due to the characteristics of the operating theater environment increase physical ergonomic risks [4, 5].

Operating theater nurses are frequently exposed to biomechanical risk factors such as heavy lifting, pushing, and pulling during work. Such activities may lead to musculoskeletal disorders. In particular, patient transfers and the use of demanding and inappropriate body mechanics related to the position of the patient in surgery cause low back and back pain. Studies have reported that operating theater nurses experience neck, shoulder, back, and lower back pain due to nonergonomic posture positions [5, 6].

The operating theater environment also poses ergonomic risk in terms of psychosocial aspects due to increased stress levels and working conditions isolated from the environment. Intense workload and long working hours due to the working conditions of operating theaters may lead to problems such as burnout syndrome and job dissatisfaction in nurses. In addition, an isolated working environment may lead to a lack of social support and a feeling of loneliness [1, 4].

Although there are ergonomics studies on many occupational groups in the literature, there are few comprehensive studies on the working positions and working environments of operating theater nurses. Existing studies have mostly focused on determining musculoskeletal system diseases (MSDs) experienced by nurses. For example, the problem of low back pain in nurses has been extensively analyzed in the literature [5, 6].

The International Commission on Occupational Health and Safety defines MSDs as “work-related musculoskeletal disorders (WMSDs),” and they are also called repetitive motion injuries. MSDs are characterized by disability, impairment, discomfort, or pain in muscles, tendons, and joints. MSDs are conditions that occur with mild and short-term pain and may require serious treatment in the future and cause dysfunction. These problems can be caused by one or more traumas affecting the musculoskeletal system. Although an organic pathology is usually not detected, it has been reported that repetitive movements, poor posture, or excessive force may cause these disorders [7–10].

In studies related with MSDs, working in nonanatomic positions, standing for long hours, working in the same position continuously, and repetitive movements were determined as ergonomic risks that may be related to these problems [11, 12]. When evaluated in terms of these risks, it is seen that operating theater nurses are under great risk in terms of MSD.

The International Council of Nurses (ICN) states that the working environment of nurses is one of the most dangerous working environments and includes risks such as occupational accidents and occupational diseases. The protection of the health and well-being of nurses in the working environment is directly related to the protection of individual, family, and community health [4].

Deterioration in the health status of operating theater nurses, who are responsible for the safety of patients during the surgical process in the operating theater, may lead to losses in work efficiency, increase in work-related accidents, pose a risk for patient and employee safety, and cause labor and financial losses [7].

In order to prevent MSD due to physical and biomechanical ergonomic risks, it is recommended to create awareness among operating theater nurses. For this purpose, this subject should be included in pregraduation and postgraduation in-service training. In addition, it is recommended that the physical conditions of the operating theater should be made ergonomic “reviewing the size and layout of the operating room, the height of the operating table should be between 70% and 80% of the elbow height and the height of the table should be adjusted according to the tallest team member and the other team members should use the stool; the height of the stool should be adjusted so that the feet are flat and comfortable on the floor during the use of the stool and to prevent back humping etc.” During intraoperative procedures, it is recommended that nurses should avoid bending forward, lying down, and staying in the same position for a long time, avoiding long-term postures involving flexion and protraction of the neck more than 20°, standing with feet hip-width apart to prevent pelvic asymmetry, and avoiding long-term continuous static posture. In addition to these recommendations, it is recommended in the literature to give rest breaks in the work program of operating theater nurses who are considered to be at high risk in terms of MSD to evaluate musculoskeletal system health regularly, to create resting areas lying on their backs in the operating theater, to provide training to gain correct body posture, and to create incentives and opportunities for them to exercise regularly [8, 9].

Studies on ergonomic risks encountered by operating theater nurses have shown that intraoperative postures and positions are important for MSDs. Within the scope of this study, it was aimed to determine the risks of body postures of operating theater nurses and working conditions in the operating theater in terms of MSDs and the effects of these risks on nurses.

## 2. Materials and Methods

The data of this study were collected from the operating room nurses of seven hospitals belonging to a private health institution between April 1 and August 31, 2021. To be included in the study, nurses were required to have worked in the operating room for more than 1 year, to have no chronic diseases, and to have no history of musculoskeletal surgery. All methods were performed following the STROBE reporting checklist.

Study data were collected with the Extended Version of the Nordic Musculoskeletal Questionnaire (NMQ-E), Rapid Entire Body Assessment (REBA), and Nurse Introductory Characteristics Form.

NMQ-E was created by a project funded by the Nordic Council of Ministers and was brought to the literature by Kuorinka et al. [10]. An extended version was prepared by Dawson et al. [11], and this edition was adapted for nurses by Pugh et al. [12]. In this study, the Turkish version developed by Alaca et al. was used [13]. NMQ-E divides the human body into 9 anatomical regions. The presence and region of pain, the onset of any pain, the hospitalization status due to pain, the status of changing duties due to pain, and any problems in that region in the last 12 months are surveyed using a diagram. In the second part, the presence of pain in the last 12 months, the last month, and the last 7 days is questioned. In the last part, the problems in performing normal functions due to the problems experienced in the last 12 months, seeing a specialist due to this problem, taking medication, and taking leave from work are questioned. The Cronbach's *α* coefficient of the scale was determined to be 0.78.

In the power analysis for the NMQ-E sample size, the sample size was calculated as 117 individuals at a confidence level of 0.95 (*α* = 0.05), when the power of the test was 0.80 (*β* = 0.20) and the prevalence was 0.50 with an acceptable sampling error of 0.10. Data collection was completed with 120 participants. Questionnaire data were collected face-to-face by the researcher.

REBA was designed by Hignett and McAtamney to provide a fast, easy, and reliable observational analysis tool for body posture while working [14]. With this method, faulty postures that may cause work-related MSDs can be detected and help determine the measures to be taken for protection. With REBA, the risk that may arise due to the movements or the position angle during the operation and the number of additional rotations/repetitions can be determined as a numerical value. The REBA risk grading consists of 5°, from 0 to 4. The degrees are separated by numerical values from 1 to 15. Ergonomic risk assessment of working conditions is based on the obtained REBA score. The risk score increases linearly with the determined numerical value. In addition, the priority for taking precautions is determined based on the risk score obtained through the evaluation. The REBA risk score assessment is shown in Table 1.

Based on the studies in the literature for REBA evaluation, the confidence level was 0.95 (*α* = 0.05), the test power was 0.80 (*β* = 0.20) in this study, and the minimum sample size was 51 based on the two-tailed tests.

**Figure 1:**
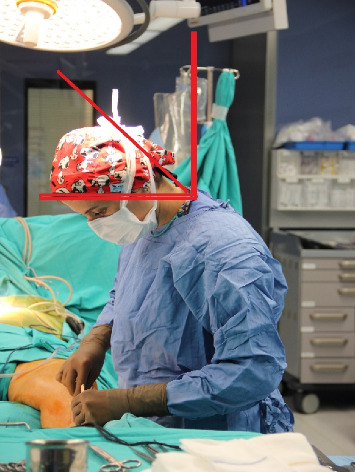
Photo sample prepared for neck assessment (graft preparation stage) (taken from the archive of the researcher).

**Figure 2:**
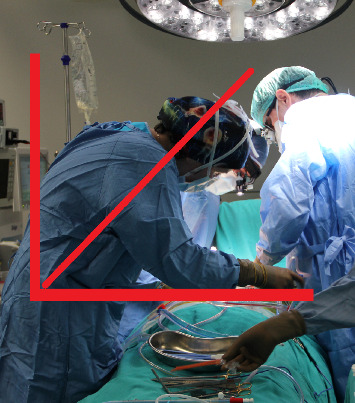
Photo sample prepared for trunk assessment (anastomosis stage) (taken from the archive of the researcher).

**Figure 3:**
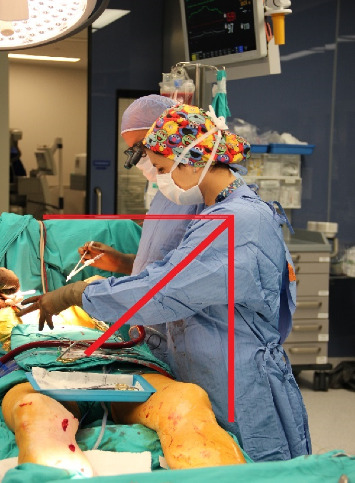
Photo sample prepared for upper arm assessment (stage of closure of the surgical field) (taken from the archive of the researcher).

Due to the type of surgical intervention and differences in surgical technique, the nurse's place in the operating room, location, and body posture in the surgical field vary. To provide standardization in the study data, data were collected from coronary artery bypass graft (CABG) surgeries performed with the traditional open surgical technique. In CABG surgeries, it is preferred that patients remain in the supine position, the surgical team and the placement of the devices used during the surgery were standardized in the surgical field, and the change in the number of teams participating in the surgery was limited. Data for the REBA assessment were obtained by intraoperative video and photo recording. By analyzing the workflow and the video records, the operation process was divided into three periods: graft preparation, anastomosis stage, and closure of the surgical field. The positions at which the nurse stayed for the longest time were determined separately for each period. The risk score was calculated by making a REBA evaluation of the determined positions. Examples of positions for which risk score calculations are made can be seen in Figures 1, 2, and 3.

The Nurse Introductory Characteristics Form was created by the researcher in line with the literature. The form questioned the factors affecting MSDs, including age, working time, working style, type of operating room, graduate school, training in ergonomics, and physical exercise status and type.

Statistical analyses of the data were performed using SPSS (IBM SPSS Statistics 24). Frequency tables and descriptive statistics were used to interpret the findings. Nonparametric methods were used for the measurement values that did not conform to the normal distribution. In accordance with nonparametric methods, the “Mann–Whitney *U*” test (Z-table value) was used to compare the measurement values of two independent groups, and the “Kruskal–Wallis H” test (*χ*^2^-table value) method was used to compare the measurement values of three or more independent groups.

## 3. Ethics

To carry out the study, written permission was obtained from Acibadem University and Acibadem Healthcare Institutions Medical Research Ethics Committee, dated 21.04.2021, and numbered 2021/08. For the “NMQ-E” used in the study, permission was obtained from the responsible author of the Turkish validity and reliability study. Permission was not required for the REBA scale since it is open to general use. Ethical rules regarding keeping the identifying information of the participants were observed. Before the application of the scales, the purpose, study plan, and objectives of the study were explained to the participants, and they were informed about the use of the obtained data and sharing it with third parties. The study was carried out with participants who stated in writing that they were willing to participate in all stages.

## 4. Findings

After the analysis of the data collected from the nurses with the introductory characteristics forms, it was determined that the mean age of the nurses was 27.9 and that 75.8% (*n* = 91) were females. In the distribution according to the total working years, those working for 1–3 years were in first place with a rate of 30.8% (*n* = 37), while those working for 6–10 years were in second place with a rate of 26.6% (*n* = 32).

It was determined that 60.8% (*n* = 73) of the nurses participating in the study worked in shifts, 53.3% (*n* = 64) worked in the operating room for more than one surgical field, 50% (*n* = 60) received undergraduate ergonomics training, and 79.2% (*n* = 95) did not receive postgraduate ergonomics training. It was reported that 71.7% (*n* = 86) of the nurses participated in sports regularly, and 14.2% (*n* = 17) participated in sports for 2-3 years. As regular sports, walking and fitness were the most common, with rates of 8.3% (*n* = 10) and 7.5% (*n* = 9), respectively.

### 4.1. Intraoperative Position–Based Risk Assessment Results

In the REBA assessment, which is used for intraoperative position–related risk assessment, 17 surgery videos and 51 photographs taken from these videos were analyzed. The work performed during the operation process was examined in 3 stages: graft preparation, anastomosis stage, and closure of the surgical field. For each stage, 17 photographs were used for measurement and analysis. After measurement and analysis, the risk score for each stage was determined. The mean risk score, total mean risk score, and standard deviation values for each stage are given in Table 2. There was a statistically significant difference (*p* < 0.05) between the risk score and surgical stages after the analysis. As shown in Table 2, the mean risk score of the anastomosis stage was higher than that of the other stages.

According to the pairwise comparisons, there was a significant difference between the anastomosis stage and both the graft preparation stage and the surgical field closure stage (*p* < 0.05), but there was no significant difference between the graft preparation stage and the surgical field closure stage (*p* > 0.05). The results can be seen in Table 3.

### 4.2. NMQ-E Results

When the NMQ-E results of the operating room nurses participating in the study were examined, it was determined that they most frequently had neck problems, at a rate of 72.5% (*n* = 87); lower back problems, at a rate of 70.8% (*n* = 85); and shoulder problems, at a rate of 65.8% (*n* = 69). Regarding the region for MSD–related hospitalization, elbows were the most common, at 11.7% (*n* = 14); the lower back was the second most common, at 6.7% (*n* = 8); and hips/thighs were the third most common, at 3.3% (*n* = 4).

The rate of nurses having difficulty performing their normal duties in their work/private lives due to MSDs was 39.2% (*n* = 47) for the lower back region, 28.3% (*n* = 34) for the neck region, and 25% (*n* = 30) for the ankles/feet.

Among the nurses who met with a physician/physiotherapist due to MSDs, neck and the shoulders were the most common areas for which treatment was sought, at 17.5% (*n* = 21); followed by the ankles/feet, at 15.8% (*n* = 19); and the upper back region, at 13.3% (*n* = 16).

It was determined that the nurses used medication for MSDs, with 42.5% (*n* = 21) using medication for the lower back region, 40.8% (*n* = 49) using medication for the neck region, and 26.7% (*n* = 32) using medication for the ankles/feet.

Regarding the rates at which nurses took time off from work due to MSDs, the rate for the lower back region was the highest, at 20% (*n* = 24); that for the shoulders was the second highest, at 13.3% (*n* = 16); and that for the neck was the third highest, at 12.5% (*n* = 15). MSDs were observed at higher rates in females than in males, but there was no statistically significant difference between the sexes (*p* > 0.05).

The incidence of MSDs according to the graduated school showed that MSDs of the neck, shoulders, upper back, elbows, hands, lower back, hips/thighs, knees, and ankles/feet were more common in those with an undergraduate degree than in the others. There was a significant difference (*p* < 0.05) between the incidence of MSDs in the neck region (46%, *n* = 40) and graduating from school.

According to the total working years of the nurses, the incidence of MSDs was higher in the neck, shoulders, upper back, elbows, hands, lower back, hips/thighs, knees, and ankles/feet for nurses who had worked for 1–3 years. In the presence of MSDs in the upper back region according to the total working time, there was a significant difference between those who had worked for 1–3 years and those who had worked for more years (*p* < 0.05).

In the distribution of MSD regions by years working in the operating room, nurses who had worked 1–3 years had high rates of MSDs in the neck, shoulders, upper back, elbows, hands, lower back, hips/thighs, knees, and ankles/feet, but these rates were not statistically significant (*p* > 0.05).

According to the working style, the incidence of MSDs in the neck, shoulders, upper back, elbows, hands, lower back, hips/thighs, knees, and ankles/feet was higher in nurses working shifts than in those who worked only during the day or night. There was a statistically significant difference (*p* < 0.05) between the presence of MSDs in the shoulders and the type of work.

MSD rates in the neck, shoulders, upper back, elbows, hands, lower back, hips/thighs, knees, and ankles/feet were higher in those working in the operating room for more than one surgical field than in those regularly working in a single surgical field, but the difference was not statistically significant (*p* > 0.05).

The nurses participating in the study who did not receive undergraduate ergonomics training had a 56.5% (*n* = 48) MSD rate in the lower back region, and there was a statistically significant difference (*p* < 0.05) between receiving ergonomics training before graduation and the presence of MSDs in the lower back region.

The rate of MSD incidence was higher for each region in nurses who did not regularly participate in sports than for those who regularly participated in sports, and there was a statistically significant difference (*p* < 0.05) between regularly participating in sports and the incidence of MSDs in the hands and hips/thighs.

A logistic regression model was created for variables with *p* values less than 0.1 in the comparison of the descriptive characteristics of nurses and MSD incidence values. As seen in Table 4, there were no statistically significant differences (*p* > 0.05) between gender, working style, total working years, regularly performed sports, and graduated school. However, the odds ratio value was 2.48 in the model established between the presence of MSDs in the lower back region and receiving ergonomics training before graduation. According to this value, MSDs are seen 2.48 times more often in those who did not receive undergraduate ergonomics training.

The odds ratio value was 1.33 in the model established between the total working years and the incidence of MSDs in the upper back region. In line with this value, it was concluded that MSDs were seen 1.33 times more often in those who had worked for 6–10 years than in others.

## 5. Results and Discussion

The results of this study show the following:

- The neck region is the most common site of WMSDs in the operating theater nurses, while the lower back and shoulder regions are the second and third most common sites of WMSDs,
- There are statistically significant relationships between the incidence of WMSDs and age, years of employment, shift work, receiving ergonomics training before graduation, and participating in sports regularly,
- Intraoperative body positions of operating theater nurses are medium/high risk for WMSDs, which puts them in the risk category that requires timely measures.

Nurses constitute the largest group among health professionals. It is known that nurses have a higher prevalence of WMSDs compared to other health professions. Low back and upper back pain are the most common among these disorders and the requirements during nursing care practices pose a risk for musculoskeletal disorders [15, 16].

Studies have shown that as the average age of nurses increases, risky movements that are not suitable for ergonomics during care practices decrease, the level of awareness increases, the ability to participate in training and practices and to exhibit positive behaviors increases. However, studies have shown that age is a disadvantage in this regard and the risk of MSD increases with age due to physical wear and being affected more quickly by poor conditions [17, 18].

Studies on the effects of working time and shift work on the incidence of MSD show that an increase in total working years, working more than one night shift, and working 40 hours or more per week are associated with poor health status, constant feeling of fatigue, and prevalence of MSD [19–21].

There is limited literature in terms of nursing studies examining the relationship between ergonomic education and the incidence of WMSDs. Few studies have been conducted to investigate the effectiveness of postgraduate education or a specific educational program, method or educational practice [17, 21]. Research on the effectiveness of ergonomics education given at the undergraduate level will be an important indicator for updating the educational content in nursing schools.

Although there is a large number of literature on the effects of sports and exercise on WMSDs, research on nurses and especially operating theater nurses is limited. The fact that nursing is a profession in which cognitive and physical studies are carried out simultaneously and physical competence is a requirement for the fulfillment of care practices makes this issue very important. In a small number of studies, it was reported that the incidence of WMSD decreased as the exercise frequency of nurses increased [22–24].

Operating theater nurses work in many postures and positions during surgery due to differences in the surgical field, surgical methods, the position of the patient during surgery, the number of surgical teams. and team placement [25, 26]. In addition, it is not possible to plan time to leave the working area, change position, or rest due to surgical asepsis conditions and risks that may arise for the patient. Intraoperative working postures of operating theater nurses should be considered as ergonomically high-risk positions [27–29]. Most of the studies on risk assessment of working posture during operations focus on surgeons, and not much attention is paid to risk assessment among nurses because of the change in position and the factors affecting it. Therefore, it is extremely important to provide recommendations for the management of ergonomic risks arising from the working conditions of operating theater nurses [30, 31].

In this study, the evaluation of ergonomic risks related to body posture in operating theater nurses shows that first performing a workflow analysis and then performing a risk assessment related to the working position of the nurse are effective.

In order for operating theater nurses to cope with working conditions and ergonomic risks arising from incorrect posture during surgeries, there are recommendations such as stress management, improvement of the equipment used, increasing the number of employees, and creating rest opportunities and muscle-strengthening exercise programs [28, 29]. Studies developing recommendations on this subject or evaluating the effectiveness of recommendations are limited. Most of the recommendations are not specific to theater nurses and are not compatible with work/posture analysis and are based on studies conducted on general nursing or health personnel. It is possible to develop more effective recommendations with studies that compare the methods used by operating theater nurses to cope with WMSDs due to ergonomic risks and these risks/negative outcomes, as well as studies supported by needs analysis.

In the literature, there are few studies showing that training programs are effective in managing ergonomic risks and preventing negative outcomes for operating room nurses [28, 32]. These studies mainly focused on postgraduate education and the effectiveness of a specific method. However, the results obtained in this study regarding the relationship between nurses who received ergonomics education at the undergraduate level and CSBs suggest that further studies on this subject will be useful in developing risk prevention recommendations.

### 5.1. Limitations

The limited number of health institutions where the study data were collected and the service conditions limit the generalization of the results to other hospitals that provide health services with different standards. The limitations of the study are the inability to compare the NMQ-E results with the REBA evaluation results since the REBA evaluation was limited to a certain type of surgery and the number of nurses working in the cardiovascular surgery operating room was insufficient to generate statistical data.

## 6. Conclusion

This study will be a starting point for more comprehensive research in the future. Studies with a high level of evidence are needed to evaluate the body postures of operating room nurses in detail in terms of ergonomic risk and to determine preventive measures for the risk score. Developing specific recommendations for operating room nurses to prevent ergonomic risks in the operating room and to protect against their negative consequences will increase the applicability of the recommendations.

### 6.1. Implications for Clinical Practice

In line with the results of this study, the recommendations to be developed with the data obtained from extended comprehensive studies should be addressed both individually for operating room nurses and institutionally for managers. In line with the results of the study, including ergonomics and ergonomic risk assessment content in the planning of the education curriculum in nursing schools will be effective in developing risk-preventive behaviors.

## Figures and Tables

**Table 1:** REBA risk score table.

Risk grading			
Degree	REBA score	Risk level	Precaution
0	1	Negligible	Not required
1	2-3	Low	May be required
2	4–7	Moderate	Required
3	8–10	High	Required in a short time
4	11–15	Very high	Required immediately

**Table 2:** REBA risk score analysis results (*n* = 51).

Stages	Mean	Standard deviation	*F*	*p* value
Graft retrieval stage	7.05	2.07	4.39	0.018
Anastomosis stage	8.7	1.5		
Surgical field closure stage	7.1	1.8		
Total	7.62	1.95		

*Note:* ANOVA.

**Table 3:** Pairwise comparison results of risk score according to post hoc analysis.

Multiple comparisons LSD						
*I* stage	*J* stage	Mean difference (*I*-*J*)	Std. error	Sig.	95% Confidence interval	
					Lower bound	Upper bound
1.00	2.00	−1.64706^∗^	0.63058	0.012	−2.9149	−0.3792
	3.00	−0.05882	0.63058	0.926	−1.3267	1.2090

2.00	1.00	1.64706^∗^	0.63058	0.012	0.3792	2.9149
	3.00	1.58824^∗^	0.63058	0.015	0.3204	2.8561

3.00	1.00	0.05882	0.63058	0.926	−1.2090	1.3267
	2.00	−1.58824^∗^	0.63058	0.015	−2.8561	−0.3204

^∗^The mean difference is significant at the 0.05 level.

**Table 4:** Logistic regression analysis results.

	**Musculoskeletal system disorder**				**Odds ratio**	**Confidence interval**		*p * **value**
	**No**		**Yes**					
	***n***	**%**	***n***	**%**				

*Region: shoulders* *Variable: gender*								
Female	64	81	27	65.85	0.46	0.19	1.1	0.08
Male	15	19	14	34.14				
Total	79	100	41	100				

*Region: shoulders* *Variable: working style*								
Day	34	43	11	26.82	0.74	0.49	1.12	0.16
Night	0	0	2	4.87				
Shift	45	57	28	68.29				
Total	79	100	41	100				

*Region: upper back* *Variable: total working years*								
1–3 Years	21	31.3	16	30.18	1.33	0.99	1.78	**0.05**
4–5 Years	11	16.4	19	35.84				
6–10 Years	18	26.9	14	26.41				
11–15 Years	5	7.5	1	1.88				
+15 Years	12	17.9	3	5.66				
Total	67	100	53	100				

*Region: lower back* *Variable: undergraduate ergonomics training*								
Yes	37	43.5	23	65.71	2.48	1.09	5.6	**0.02**
No	48	56.5	12	34.28				
Total	85	100	35	100				

*Region: hips/thighs* *Variable: regular exercise*								
Yes	6	15	28	35	0.64	0.21	1.98	0.4
No	34	85	52	65				
Total	40	100	80	100				

*Region: neck* *Variable: graduated school*								
Health Vocational High School	20	23	13	39.39	1.43	0.92	2.23	0.1
Associate degree	15	7.2	3	9.09				
Undergraduate degree	40	46	17	51.51				
Graduate degree	12	13.8	0	0				
Total	87	100	33	100				

*Note:* Statically significant *p* values < 0.05 are shown in bold.

## Data Availability

The data that support the findings of this study are available from the corresponding author upon reasonable request.

## Supporting Information

All methods were performed following the STROBE reporting checklist.
